# Using Generalizability Theory and Many-Facet Rasch Model to Evaluate In-Basket Tests for Managerial Positions

**DOI:** 10.3389/fpsyg.2021.660553

**Published:** 2021-07-29

**Authors:** Guangming Li, Yuxi Pan, Weijun Wang

**Affiliations:** ^1^Key Laboratory of Brain, Cognition and Education Sciences, Ministry of Education (South China Normal University), Guangzhou, China; ^2^Key Laboratory of Mental Health and Cognitive Science, School of Psychology, Center for Studies of Psychological Application, South China Normal University, Guangzhou, China; ^3^Department of Psychology and Clinical and Research Institute on Addictions, School of Nursing, University at Buffalo, State University of New York, Buffalo, NY, United States

**Keywords:** generalizability theory, many-facet rasch model, in-basket test, enterprise, personnel assessment

## Abstract

**Purpose:** This study aimed to analyze interview data collected from a series of in-basket tests during managerial personnel recruitment in a local Chinese company, taking advantage of the use of combination of Generalizability theory (GT) and Many-facet Rasch Model (MFRM), rather than the Classical Test Theory (CTT).

**Design/Methodology/Approach:** Participants included 78 candidates (*M*_age_ = 37.76, SD = 6.36; 35.9% female) interviewed for eight managerial positions in a local Chinese company. Data were collected based on a series of 10 in-basket interview tests, and a 20-item rating scale (the in-basket test rating scale; IBTRS) was developed and piloted, and five expert raters rated the participants on their performance in five aspects (planning, communication and coordination, capital operations and management, analysis and problem solving, and empowerment and controlling). The data were analyzed using a crossed design of *p* × *i* × *r*, where *p* represents person, *i* represents item, and *r* represents rater. Effects of candidate (person), test item, rater, and the interaction of item and rater were examined.

**Findings:** The use of the combination of GT and MFRM was able to provide accurate, comprehensive information over the process of in-basket interview tests. Specifically, GT analysis showed good generalization coefficient and reliability index (0.893 and 0.871, respectively), and the variation of candidates explained most of the total variance (53.22%). The candidates scored high in the dimension of empowerment and controlling. There were differences in the severity of raters. Three raters should be sufficient to ensure good scoring stability.

**Originality/Value:** This study used the combination of GT and MFRM to assess the interview data instead of using a CTT approach.

## Introduction

The importance of personnel assessment (the assessment under the background of human resource management) has been increasingly recognized in the practice of personnel recruitment in any successful enterprise. Assessment of personnel often includes resume analysis, paper-and-pencil tests, psychological assessment, scenario simulation exercises, and structured interviews (Pulakos et al., 1996). Using a scenario simulation method, candidates are placed in a practical situation or process during the interview so that the raters (judges) can assess related competencies and abilities of candidates through verbal communication and behavior observation. A scenario simulation method often includes two major forms, leaderless group discussion and in-basket tests. A series of well-designed in-basket test interviews could be an optimum strategy for selecting enterprise leaders (e.g., general managers).

In-basket test is a kind of evaluation form which is widely used in assessment center technology. As a scenario simulation technology to evaluate the quality of middle and senior managers, in-basket test has been studied and applied for more than 70 years. Since 1957, many internationally well-known companies have carried out the research and application of the in-basket test. For example, in the United States, the test has been adopted by more than 1,000 well-known enterprises, such as AT & T, Ford Motor, and General Electric (Song et al., 2008). The in-basket test has been taken as an important method of selecting and evaluating enterprise managers. In China, the research and application of in-basket test started later, but as an effective selection tool, it has received more and more attention and is increasingly used in the recruitment and selection of national leading cadres and managers (Song et al., 2008). According to Peng and Wang (2006), the frequency of using in-basket test in practice accounts for 89% of the assessment center method, next only to general face-to-face interview (93%).

In a typical in-basket test, examinees are placed in the simulation situation of a specific management position and are provided a batch of documents that the position often needs to deal with, such as memos, emails, letters, and calendars. Examinees should respond to each document within specified time and conditions, including prioritizing tasks, finding mistakes in expense vouchers, and determining how to handle a problem employee (Whetzel et al., 2014). After evaluation, according to pre-designed scoring dimensions and standards, raters will evaluate and rate the various abilities of the examinees in the process of the test, such as planning ability, analysis and judgment ability, and decision-making ability (Brass and Oldham, 1976). However, several factors should be considered in the personnel assessment process. These factors could be the presence of subjective opinions of raters, the familiarity of test situation, test items, ability and performance of a candidate, and interview evaluation criterion for raters. Consideration of these variables could reduce in-basket test scoring errors and maximize reliability and validity. In recent years, the Classical Test Theory (CTT) has been widely used in interview studies. Interviews like in-basket tests should be evaluated at both the macro and micro levels. In that regard, Generalizability theory (GT) and Many-facet Rasch Model (MFRM) have advantages, especially when these two methods are combined, relative to CTT (Iramaneerat et al., 2007). For example, from a macroscopic perspective, GT can be used to identify various sources of error that may affect measurement target, based on influencing factors (Lee and Park, 2012). Using different designs, researchers can use GT to estimate these errors and identify favorable information in decision-making and appropriate approaches to general control of test designs (Spooren et al., 2014). At the same time, researchers can use MFRM to analyze the internal information of a test from a microscopic perspective and control errors. MFRM, as an Item Response Theory (IRT) Model, has the advantages of microscopically evaluating the difficulty of test items, the ability of a candidate, severity of raters, and consistency of scores on the same scale. Thus, candidates can be distinguished from one another with different abilities, and all facets of the in-basket test can be identified (Wang, 2003). GT and MFRM can be combined to analyze an in-basket test and model fit improvement solutions could also be provided. Using the combination of GT and MFRM could be a more conducive statistical option for an enterprise to fully and scientifically use results of in-basket test assessment data, select appropriate candidates, improve interview routines, and train raters (Kozaki, 2004).

Generalizability theory, as an important psychometric theory, has been developed on the basis of CTT. GT is firmly established in mainstream statistics, and its use is increasing in various enterprises and governmental and educational evaluations (Oghazi, 2016). GT includes generalizability study (G study) and decision study (D study) (Pleschov and McAlpine, 2016). G study could be viewed as a development process of measurement routines and aims to find out various potential sources of measurement error and estimate their variance components. In this study, we examined three facets (i.e., sources of variation): raters, test items, and candidates (persons). G study describes the main effects, interaction effects, and errors in terms of variance components, which reflect the relative effect of each facet (Dogan and Uluman, 2017). Cross-design treats persons (p; candidates in this study) as measurement targets and test items (i) and raters (r) as measurement facets. The formula for G study can be described as:

(1) $$\begin{array}{r} X_{pir}=\mu +V_{p}+V_{i}+V_{r}+V_{pi}+V_{pr}+V_{ir}+V_{pir,e} \end{array}$$

where μ represents the population mean; *V*_*p*_, *V*_*i*_, and *V*_*r*_ represent the effects of persons, test items, and raters, respectively; *V*_*pi*_, *V*_*pr*_, and *V*_*ir*_ are the interaction term effects of persons and test items, of persons and raters, and of test items and raters, respectively; and *V*_*pir,e*_ is the residual effects.

Decision study involves converting and interpreting test scores and could be considered as an application of measurement routines. D study intends to reduce measurement error and improve reliability according to decision-making and estimates of variance components of G study (Linacre and Wright, 2002). Cross-design treats persons (p) as measurement targets, and test items (I) and raters (R) as measurement facets. The formula for G study can be written as:

(2) $$\begin{array}{r} X_{pIR}=\mu +V_{p}+V_{I}+V_{R}+V_{pI}+V_{pR}+V_{IR}+V_{pIR,e} \end{array}$$

Generalizability theory shows an important statistical framework for not only identifying factors that affect the reliability of measurements but quantifying their influence on the dependability of the scoring (Cronbach et al., 1972). Results of GT can improve the confidence of examinees in their in-basket measurements and can help make informed decisions about how measurements might be better taken in subsequent evaluation efforts. In contrast to MFRM that produces individual-level estimates of components adjusted for all other facets, G-theory analysis usually provides a group-level overview about relative contributions of all the facets (Zhang and Roberts, 2013). In conclusion, GT is a useful method for monitoring the quality of an in-basket test, which can distinguish the source of measurement error of different facets of the assessment, so that the findings from the GT analyses can also lead to recommendations for improving the quality of the in-basket test. Rasch concentrates on the individual examinee. For each examinee, a measure is estimated that is as independent as is statistically possible for the particularities of the raters, items, tasks etc, that the examinee encountered (Linacre, 1993). MFRM has been developed on the basis of one-parameter Rasch model in IRT. The one-parameter Rasch model estimates the difficulty of items and competence of candidates; however, it also allows researchers to estimate the design of tasks and severity of raters, and to assess the combination of factors that may not match each other using bias analysis (MacMillan, 2000). The formula for MFRM can be described as follows:

(3) $$\begin{array}{r} \log\left(\frac{P_{nijk}}{P_{nij\left(k-1\right)}}\right)=B_{n}-D_{i}-C_{j}-F_{k} \end{array}$$

where *P*_*nijk*_ is the probability of candidate *n* rated *k* by judge *j*on item *i*; *P*_*nij*(*k*−1)_ is the probability of candidate *n* rated *k* − 1 by judge *j* on item *i*; *B*_*n*_ represents the performance measure of candidate n (*n* = 1, 2,., *N*); *D*_*i*_ represents the difficulty of item *i* (*i* = 1, 2,., I); *C*_*j*_ represents the severity of judge *j* (*j*= 1, 2,., *J*); and *F*_*k*_ represents the difficulty of rating step [category] *k* relative to rating step [category] *k* − 1 (*k* =1, 2,., *K*).

Many-facet Rasch Model concerns itself with obtaining from raw ratings of each examinee a linear measure corrected for the particular raters or tasks that the examinee encountered (Iramaneerat et al., 2007), allowing us to identify particular elements within a facet that are problematic, or “misfitting” (Lynch and McNamara, 1998). In the process of statistical analysis, MFRM can eliminate the influence of specific items and rater biases to get the ability value of a candidate, which is independent of the difficulty of specific items and characteristics of raters. Therefore, based on the MFRM analysis, the decision-making of assessment will be more objective and fairer. In addition, MFRM can provide the degree of leniency and strictness in the scoring process of different raters, which shows the impact of the rater effect on scoring more intuitively, helps raters identify unqualified raters, and improves the accuracy of evaluation results. Finally, through deviation analysis, using MFRM can quickly and effectively distinguish the “problem” and unqualified examinees and raters so that effective measures (such as reevaluation, replacement or training of raters) can be taken to ensure the quality and overall consistency of scoring.

To illustrate the use of these two diverse, but complementary, methods to estimate the quality of in-basket test, the research analyzed scores from managerial personnel recruitment in a local Chinese company. We used GT for two major purposes (1) to identify the major sources of measurement error and (2) to arrange the sample sizes of raters and items for in-basket test practice. We used MFRM to find (1) the distribution of the ability of candidates, severity of raters, and difficulty of test items; and (2) the bias effects of raters and candidates and identify inconsistency between the evaluation of raters.

## Method

### Sample and Procedure

The participants included 78 candidates (*M*_age_ = 37.76, SD = 6.36; 35.9% female) interviewed for eight general manager and vice general manager positions at a local company in a medium-sized metropolitan area in Guangzhou, China. Ads were published on the official website of the city government; 234 applicants were screened, and 78 (33.3%) applicants who had prior management experience were interviewed using a series of 10 in-basket tests (i.e., 10 documents; see Appendix C; see Appendix D, the Chinese version of in-basket tests). We piloted a self-developed 20-item rating scale using a pilot sample to measure five dimensions of the abilities of a candidate: planning, communication and coordination, capital operations and management, analysis and problem-solving, and empowerment and controlling (see below). Each dimension consisted of four items. The raters were five people (*M*_age_ = 51.5; two females) who had rich knowledge of and work experience in business management and psychology fields. All the participants and raters provided informed consent. All study procedures were approved by the University Research Ethics Board of South China Normal University (Institutional Review Board). First, the interviewer gave out the test materials and answer book and put forward the precautions for the test to the participants, including the closing of the communication equipment, confirmation of the test materials and answer book, and filling in of personal information of the participants. The test materials included background information about industries, enterprises, departments, and task documents. The information on background materials could help them to better understand the basic knowledge of their responsibilities and opportunities. Task documents were the core of the basket test, which could be divided into three types: review documents, decision-making documents, and perfect documents. Review documents are generally routine official documents, which mainly require the participants to deal with tasks step by step, distinguish the priority of documents, and put forward corresponding treatment opinions. Decision-making documents include requests, reports, and suggestions, which are from the lower level of management or from the outside of the organization. The contents described are generally unconventional decision-making problems encountered in typical daily work. The main requirements are that the participants put forward a decision-making scheme after comprehensive analysis of the documents or choose the best scheme among the existing schemes. Perfect documents mainly refer to the documents that lack certain conditions and information, such as incomplete materials and improper views, which mainly require the participants to put forward corresponding problems, further obtain information, and solve problems. The participants then tried to create a realistic management situation through guidance language and independently completed their own basket materials. Finally, after the reversion test of the first defense and modification of their basket materials, the assessor gave a score and evaluation one by one according to the processing of each document and each dimension to be investigated and compared the answers of the evaluated with the reference answers made in advance. The final score was not simply the sum of the scores of each dimension but a comprehensive evaluation of the overall performance of a participant.

### The In-Basket Test Rating Scale

The rating scale, IBTRS, included 20 items, on a five-point scale, ranging from 1 (strongly disagree) to 5 (strongly agree) (see Appendix A; see Appendix B, the Chinese version of the scale). We analyzed the IBTRS using a series of exploratory factor analysis (Geiser, 2012) in a pilot sample (*n* = 318, *M*_age_ = 39.46, SD = 6.24; 42.1% female) prior to this research. We summed all four items under each of the five dimensions. The model fit was acceptable (Schermelleh-Engel et al., 2003): CFI/TLI = 0.901/0.912, RMSEA = 0.056 [95% CIs, 0.043, 0.067], and SRMR = 0.044. Internal consistency for the overall scale (i.e., all 20 items) was α = 0.92. Internal consistency for the subscales was good: planning α = 0.88; communication and coordination α = 0.83; capital operations and management α = 0.90; analysis and problem solving α = 0.84; and empowerment and controlling α = 0.92. The correlations among these dimensions were 0.51 < *r* < 0.65 (Table 1).

**Table 1:** Minimums, maximums, means, standard deviations, and correlations for five dimensions.

**Descriptive statistics**						**Correlations**				
**Variable**	***N***	**Min**	**Max**	**Mean**	**SD**	**1**	**2**	**3**	**4**	**5**
1. Planning	78	5.00	20.00	16.08	3.07	–				
2. Communication and coordination	78	5.00	20.00	15.53	3.05	0.58^**^	–			
3. Capital operations and management	78	4.00	20.00	16.58	3.69	0.56^**^	0.61^**^	–		
4. Analysis and problem-solving	78	4.00	20.00	14.80	3.82	0.57^**^	0.65^**^	0.64^**^	–	
5. Empowerment and controlling	78	4.00	20.00	15.11	3.68	0.69^**^	0.61^**^	0.62^**^	0.63^**^	–

*1 = Planning, 2 = communication and coordination; 3 = capital operations and management, 4 = analysis and problem solving, 5 = empowerment and controlling*.

** *Correlation is significant at the 0.01 level (2-tailed)*.

### Statistical Strategy

The interview data were analyzed using GT and MFRM. A crossed design of *p* × *I* × *r* was used in this GT study. We conducted GT analysis with the computer program GENOVA (Version 3.6; Linacre, 2007) and MFRM analysis with the computer program FACETS (Version 3.5; Crick and Brennan, 1983). Specifically, in the crossed design of *p* × *i* × *r*, i represents the total score of each dimension, which is equal to the summed score of the four items of each dimension, not a single item score. That is, *i* denotes the dimension score.

## Results

The minima, maxima, means, SDs, and correlations for the five dimensions of our evaluation are presented in Table 1.

### Generalizability Theory

#### Generalizability Study

The pattern of the results from G study is shown in Table 2. Persons explained the largest percentage of the total variance (53.22%), which indicates that in-basket tests can distinguish the abilities of candidates to a certain degree. The interaction effects of persons and items (pi) explained the second largest percentage of the total variance (30.09%). The candidates responded differently to the interview question items. Items (4.23%) and raters (3.22%) also contributed to the variability of test scores (performance). There was inconsistency in the difficulty of items and severity of raters. Residual effects accounted for 9.24% of the total variance.

**Table 2:** G study.

**Effect**	**df**	**MS**	**Variance component**	**Percentage of**
				**total variance (%)**
Persons (p)	77	73.19	2.61	53.22
Items (i)	4	89.09	0.21	4.23
Raters (r)	4	62.08	0.16	3.22
pi	308	7.84	1.48	30.09
pr	308	0.45	0.00	0.01
ir	16	0.41	0.00	0.02
pir,e	1,232	0.45	0.45	9.24

#### Decision Study

Results from D study are displayed in Table 3. The generalizability coefficients (G-coefficients) and dependability coefficients associated with various combinations of raters and items are provided. The variance component of persons remained unchanged (2.614), regardless of the conditions of items and raters. When the number of raters was fixed, increases in the number of items (e.g., from 3 to 7) led to gradual increases in G-coefficient and dependability coefficient values, indicating that an appropriate increase in the number of raters may improve the reliability of test scores. Specifically, the largest increase in G-coefficient values emerged when the number of items increased from 3 to 4.

**Table 3:** D study.

**Raters**	**Items**	**Variance component of persons**	**Norm-referenced Test**	**Criterion-referenced Test**
			**G-coefficients**	**Dependability coefficients**
5	7	2.61	0.92	0.90
4	7	2.61	0.92	0.90
3	7	2.61	0.92	0.89
2	7	2.61	0.92	0.88
1	7	2.61	0.90	0.85
5	6	2.61	0.91	0.89
4	6	2.61	0.91	0.89
3	6	2.61	0.91	0.88
2	6	2.61	0.90	0.87
1	6	2.61	0.89	0.84
5	5	2.61	0.89	0.87
4	5	2.61	0.89	0.87
3	5	2.61	0.89	0.86
2	5	2.61	0.89	0.85
1	5	2.61	0.87	0.82
5	4	2.61	0.87	0.85
4	4	2.61	0.87	0.84
3	4	2.61	0.87	0.84
2	4	2.61	0.86	0.82
1	4	2.61	0.84	0.79
5	3	2.61	0.83	0.81
4	3	2.61	0.83	0.80
3	3	2.61	0.83	0.80
2	3	2.61	0.82	0.79
1	3	2.61	0.80	0.75

When the number of test items was fixed, G-coefficient values and dependability coefficients also gradually increased when the number of raters increased from 1 to 5, indicating that an appropriate increase in the number of test items may improve the reliability of test scores. Results showed that the largest increase in the dependability coefficient appeared when the number of raters increased from 1 to 2. When the number of raters was three or more, increases in dependability coefficients became small. Thus, the stability of test scores could be maintained with three raters. A further increase in the number of raters would not significantly improve the stability, which is consistent with the findings of Lakes (2013).

#### Many-Facet Rasch Model

**Figure 1:**
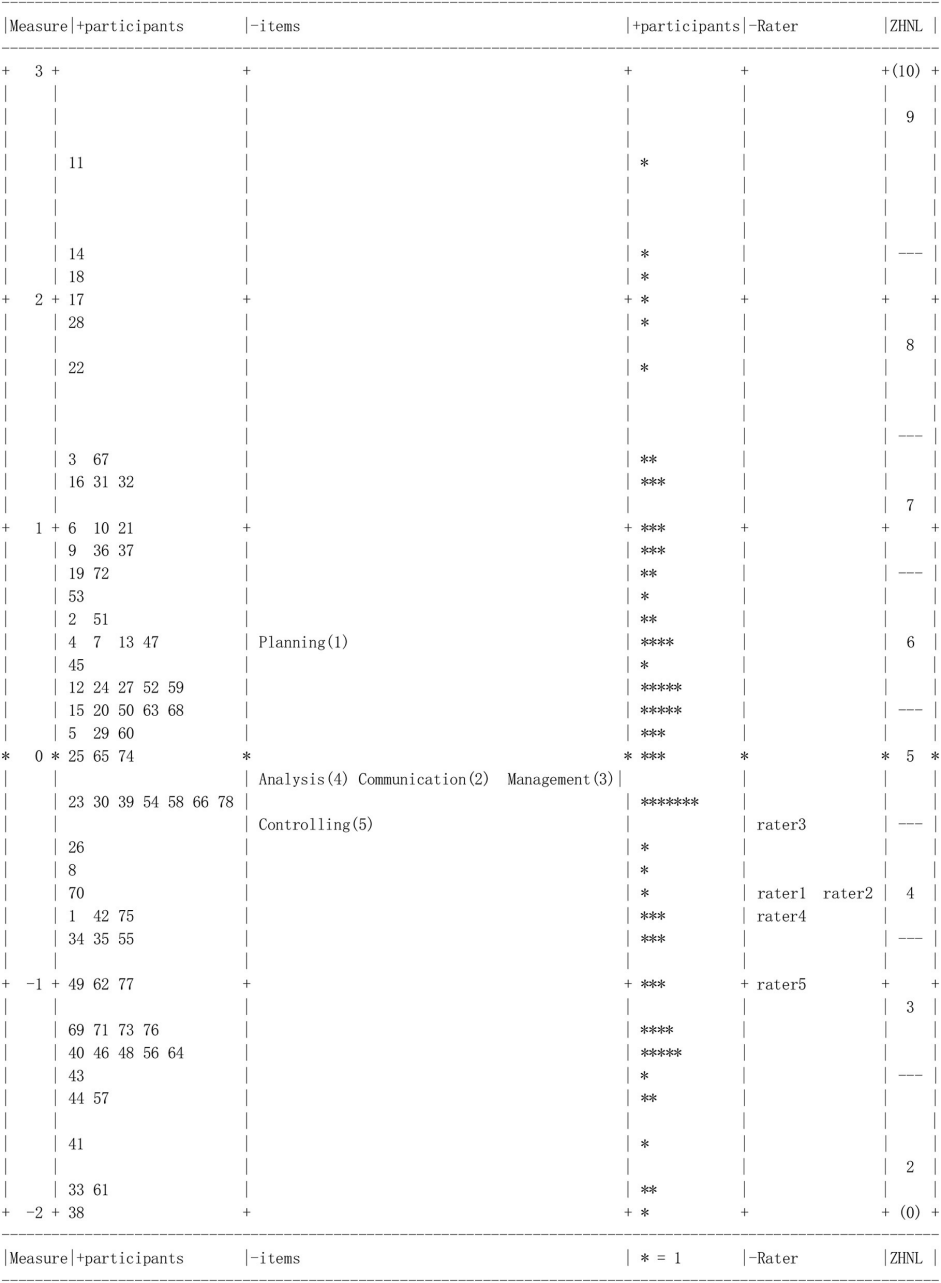
Variable map of all facets.

The distribution of the ability of candidates, severity of raters, and difficulty of test items is displayed in Figure 1. “Measure” on the left column of Figure 1 represents the number of logit units. Computer program FACETS was used to analyze all facets in logit units. As shown in Figure 1, the ability of candidates (left column), difficulty of test items, and severity of raters are ranked from top to bottom, respectively. In the left column, numbers 1–78 represent individual candidates. The ability distribution of candidates was largely concentrated within the range of ±1 logit. Candidate number 11 ranked highest and candidate number 38 ranked lowest in the ability of candidates. On the right side are difficulty evaluation of test items and severity evaluation of interviewers, which are also arranged from top to bottom according to the difficulty of test items and the severity of interviewers. The severity distribution of raters was also relatively small, indicating consistency across raters while they score the performance of the candidates in the in-basket tests. The item difficulty distribution was relatively small. The empowerment and controlling dimension was relatively easy for the current sample.

The right column of Figure 1 is the score segment representing the expected average score value, which can be understood by corresponding it to the column of “candidate's ability estimation.” This column aims to display the distribution of the expected average score of the candidate, such as 5–7, which is the score segment that most candidates got. At the same time, this column can also reflect the differences in the expected score of each candidate: the expected score of candidate number 11 was the highest, reaching above nine; the expected score of candidate number 38 was the lowest, only about two. The parameter estimation of the expected average score was also presented in the measurement report of the candidates generated by the FACTS software. For example, the expected average score of the actual number 11 candidate was 9.3.

#### Candidate Analysis

Many-facet Rasch Model results on the facets of the candidate are shown in Table 4. Candidate ability values ranged from −1.99 logits (candidate number 38) to 2.58 logits (candidate number 11). The separation reliability was 0.98. There was no central tendency for test scores. The in-basket test indicated that the degree of inconsistency among the candidates was 98%. It means that the distribution of interview scores of these candidates does not show a centralized trend. The candidates were significantly different in their ability, χ^2^(df) = 2,864 (77), *p* < 0.001. The Infit MS reflects the fitting of the ability assessment of a candidate and the consistency of the scoring of raters. If the Infit MS of a candidate is larger than two SDs, the rating consistency between the raters will be poor. In this study, the average Infit MS value was 0.99, and SD was 0.84. Therefore, the rating consistency would be good if the Infit MS was <2.67. As shown in Table 4, the Infit MS of four candidates (i.e., numbers 40, 44, 53, and 63) was higher than 2.67. There was poor consistency among the five raters in rating these four candidates, especially, in candidate number 40, where the Infit MS was as high as 4.57.

**Table 4:** FACETS analysis of the ability of candidates.

**Participant**	**Ability**	**Error**	**Infit MS**	**Participant**	**Ability**	**Error**	**Infit MS**	**Participant**	**Ability**	**Error**	**Infit MS**
1	−0.72	0.15	1.41	27	0.32	0.16	1.02	53	0.66	0.17	3.86
2	0.60	0.16	0.40	28	1.95	0.21	2.13	54	−0.25	0.15	1.46
3	1.31	0.19	0.52	29	0.11	0.15	1.53	55	−0.82	0.15	0.22
4	0.50	0.16	0.56	30	−0.25	0.15	1.28	56	−1.26	0.15	0.72
5	0.11	0.15	0.74	31	1.20	0.18	0.87	57	−1.48	0.15	1.61
6	1.04	0.18	0.78	32	1.24	0.19	0.56	58	−0.18	0.15	0.59
7	0.47	0.16	0.64	33	−1.87	0.16	0.25	59	0.27	0.15	1.21
8	−0.55	0.15	0.86	34	−0.82	0.15	0.23	60	0.11	0.15	0.51
9	0.91	0.17	0.57	35	−0.80	0.15	1.32	61	−1.87	0.16	0.39
10	1.01	0.18	0.91	36	0.85	0.17	0.48	62	−0.95	0.15	0.42
11	2.58	0.25	0.72	37	0.91	0.17	0.78	63	0.25	0.15	3.64
12	0.35	0.16	0.34	38	−1.99	0.16	0.37	64	−1.34	0.15	0.27
13	0.55	0.16	0.41	39	−0.20	0.15	0.41	65	0.02	0.15	1.37
14	2.24	0.23	0.63	40	−1.32	0.15	4.57	66	−0.20	0.15	0.63
15	0.20	0.15	1.12	41	−1.70	0.15	0.59	67	1.27	0.19	0.46
16	1.24	0.19	1.36	42	−0.67	0.15	2.48	68	0.23	0.15	0.66
17	2.04	0.22	1.63	43	−1.41	0.15	0.24	69	−1.23	0.15	0.26
18	2.13	0.22	1.24	44	−1.50	0.15	3.39	70	−0.61	0.15	0.84
19	0.83	0.17	0.73	45	0.45	0.16	0.46	71	−1.19	0.15	0.20
20	0.25	0.15	0.39	46	−1.26	0.15	0.21	72	0.77	0.17	0.79
21	0.98	0.18	0.93	47	0.55	0.16	0.46	73	−1.19	0.15	0.37
22	1.69	0.20	0.79	48	−1.28	0.15	1.18	74	−0.05	0.15	2.21
23	−0.22	0.15	1.79	49	−1.00	0.15	1.40	75	−0.67	0.15	1.27
24	0.27	0.15	0.65	50	0.23	0.15	0.76	76	−1.21	0.15	0.55
25	−0.05	0.15	0.41	51	0.63	0.16	0.58	77	−0.95	0.15	1.44
26	−0.37	0.15	0.99	52	0.32	0.16	1.52	78	−0.16	0.15	0.50

*Separation reliability = 0.98, chi-square = 2,864, df = 77, significance = 0.001, Infit SD = 0.84*.

#### Items Analysis

Many-facet Rasch Model results on the item facet are shown in Table 5. Items analysis focused on difficulty, difficulty differences, and fitting degree of the five dimensions of ability of candidates. The difficulty of empowerment and controlling was the lowest (−0.26 logits). The scores of the candidates on empowerment and controlling were relatively high. The item separation reliability reflects the difference in difficulty among the five dimensions. The results showed that the item separation reliability was 0.98. There were differences in item difficulty in these five studied dimensions, but these differences did not reach the significant level (*p* = 0.27). Infit MS is shown in the right column (Table 5). The average Infit MS of the five dimensions was 0.98, and SD was 0.68. Thus, there would be a good difficulty consistency if the Infit MS ranged from −0.38 to 2.34. As shown in Table 5, all the five Infit MS values fell in this range, and the fitting degree of in-basket test items was good.

**Table 5:** FACETS analysis of item difficulty.

	**Item**	**Difficulty**	**Error**	**Infit MS**
1.	Planning	0.50	0.04	2.17
2.	Communication and coordination	−0.12	0.04	0.56
3.	Capital operations and management	−0.05	0.04	0.89
4.	Analysis and problem–solving	−0.07	0.04	0.61
5.	Empowerment and controlling	−0.26	0.04	0.65

*Separation Reliability = 0. 98, chi-square = 3.9, df = 4, significance = 0.27, Infit SD = 0.68*.

#### Rater Analysis

Many-facet Rasch Model results on the rater facet are shown in Table 6. Rater analysis mainly tested the rationality of the rating of raters from two aspects: severity and internal consistency. As shown in Table 6, among the five raters, rater number three was most severe (−0.33 logits), and rater number five was relatively loose (−1.01 logits). In addition, the separation reliability was 0.79. There were severity differences among the raters χ^2^(df) = 154.1 (4), *p* < 0.001.

**Table 6:** FACETS analysis of the severity of raters.

**Rater**	**Severity**	**Error**	**Infit MS**
1	−0.57	0.04	0.88
2	−0.55	0.04	1.03
3	−0.33	0.04	0.91
4	−0.73	0.04	1.16
5	−1.01	0.04	1.01

*Separation reliability = 0.79, chi-square = 154.1, df = 4, significance = 0.001, Infit SD = 0.11*.

Infit MS values (the right column of Table 6; a weighted mean square statistic) reflect the internal consistency of the rating raters. If the Infit MS value is =1, the model fits the data well. If the Infit MS is ±2 SDs above the mean, the consistency within the raters will be poor, and the raters should be retrained or replaced to ensure reliability. In this study, the average value of the Infit MS was one, indicating that the rating of raters was consistent overall. The SD was 0.11. The Infit MS should range between 0.78 and 1.22 in order to receive good internal consistency. As shown in Table 6, all the five Infit MS values were within this range, and there was consistency in the rating of raters.

#### Bias Analysis

Bias analysis is intended to show the bias effects of raters and candidates (390 pairs in total) and identify the inconsistency (if any) between the evaluation of raters (Table 7). If T > 2, the candidate is rated too leniently; whereas if T < −2, the candidate is rated too severely. As displayed in Table 7, there was a significant deviation effect (T < −2), and rater number 2 rated candidate number 21 too severely.

**Table 7:** Bias analysis of raters and candidates.

**Rater**	**Participant**	**Assessment ability**	**Expected ability**	**Bias**	**SE**	**T**
2	21	0.52	1.80	−1.28	0.61	−2.08

## Discussion

### Use of a Combination of Generalizability Theory and Many-Facet Rasch Model

We analyzed a small sample of 78 candidates interviewed for eight managerial positions in a local Chinese company. The combination of GT and MFRM was used, and facets of candidates, items, raters, and interactions were examined on their performance in a series of in-basket interview tests. The variance in candidates (persons) accounted for the largest amount of the total variance in G study (53.22%). Although these facets explained most of the total variance, the residual effect accounted for 9.24% of the total variance. Future studies should consider other possible contributing factors (facets). D study provided the generalizability coefficients and reliability index, and the results (Table 3) were relatively ideal from the perspective of GT. The literature appears to indicate that the ideal situation is when the generalization coefficient and reliability index exceed 0.9. The series of in-basket tests was able to distinguish between candidates in their performance. However, the MFRM analysis from a micro perspective showed that the inconsistency in the rating of raters was a source of variation. There were differences between (and within) the raters in rating the candidates. For example, the raters disagreed on rating the ability and performance of several candidates (e.g., candidates number 40, 44, 53, and 63). Relevant training programs should be in place for the raters. The D study showed that the reliability index remained stable when the number of raters was up to three. Three raters could ensure good scoring stability (Lakes, 2013). We used MFRM to examine individual raters. Although the fitting degree of evaluation within the raters was good, the severity between raters was significantly different. The reliability indexes of three raters were close to those of five raters. The two raters with a large difference in severity could withdraw from rating in subsequent interviews. This could control rater differences and also save manpower and material resources.

G study showed that there was room for improvement in the design and selection of test items. D study indicated that, when the number of test items reached seven, the reliability index reached 0.9, and increases in test items also improved the reliability index. It may be possible that the number of items could be increased to improve reliability, or existing items could be subdivided. For example, the dimension of analysis and problem-solving ability or the dimension of empowerment and controlling ability could be divided to improve the reliability of test results. However, this is beyond the scope of this study. Future studies should investigate this possible division method.

The use of the combination of GT and MFRM in the in-basket tests dealt with every single facet (i.e., candidate, test item, and rater) and the interaction effects between these facets. GT provided descriptive information about each facet and predictive information about the number of test items and raters. MFRM verified the results produced from GT, identified the sources of variance difference one by one, and provided feedback information on all the facets, such as the reliability index and chi-square values. Researchers are encouraged to use these information sources in the practice of human resources, such as personnel selection and training and improvement of related evaluations. The interaction effects of GT and MFRM should also be helpful in confirming the interview results. For example, the results showed that the interaction effects between candidates and raters were relatively small (Table 2). The bias analysis in MFRM showed that only one out of the 390 pairs of data from the candidates and raters had a significant difference (Table 7). This confirmed our finding in G study that there was an interactive effect, but that effect was relatively small.

How could human resource management practitioners apply our approaches to evaluate their own in-baskets test? Before launching the in-basket test, human resource professionals should follow the recommendations of previous in-basket generalizability studies and arrange the number of test questions and interviewers. For example, in this study, we found that when the number of test items increased from three to four, the increase in generalizability coefficient was the largest, and three interviewers could ensure good scoring stability. Based on this, human resource practitioners can make a comprehensive consideration according to the recruitment requirements and interview screening mechanism of the company. Second, before the test, human resource professionals should conduct unified training for interviewers, unify evaluation criteria, and take measures to reduce error from the in-basket interview. After the test, human resource practitioners can use the generalizability method to test the reliability of the test. They can also use MRFM to detect the severity of several interviewers, the difference in the ability of candidates, and difficulty of test items, and get the severity of the evaluation of an interviewer and the level of the ability of candidates so as to adjust the score accordingly. We expect that different raters have different degrees of leniency. When there are significant deviations in the consistency between raters, it would be difficult to effectively distinguish the competencies of candidates from one another. Once we know that a rater is strict or loose, appropriate statistical methods should be considered to reduce the evaluation weight of a rater. When the rater has a large proportion of deviation in the deviation analysis between raters and participants, we have reason to doubt whether there is a Halo effect or cheating. The use of these technologies may require measurement knowledge and skills of human resource professionals, which may indicate why the combination of two methods has been rarely used in practice.

### Limitations

The sample in this study was relatively small. Unfortunately, the sample size was limited by the interview screening mechanism. We used a series of 10 documents as in-basket interview tests, but it was unknown whether these 10 tests were sufficient to capture the abilities of the candidates. In the crossed design model (*p* × *i* × *r*), we ignored a single item score; rather, we considered the total score by summing the four items for each dimension. Also, the designs, such as *p* × *(i: d)* × *r*, could be modeled, where *i* could represent item and *d* could represent dimension. The IBTRS is a newly developed scale to assess the performance and ability of candidates. Although we conducted a series of exploratory factor analyses using an independent, pilot study sample, we were short of external validity evidence for the rating scale. Future studies should address this issue.

### Implications and Conclusions

We used GT and MFRM to assess a series of in-basket tests in a small sample of 78 candidates interviewed for managerial positions in a local Chinese company. GT analyzed the impact of candidates, test items, and raters on test scores from a macro perspective. GT analysis showed good generalization coefficient and reliability index (0.893 and 0.871, respectively). The variation in candidates explained most of the total variance. When the number of test items increased from three to four, the generalization coefficient increased most; three raters should be sufficient to ensure good scoring stability. MFRM, from a micro perspective, examined the difference between the ability of candidates, difficulty of test items, and severity of raters. Using the combination of GT and MFRM could provide accurate and comprehensive evaluation information and results on enterprise (and other organizations) in-basket tests.

## Data Availability Statement

The original contributions presented in the study are included in the article/Supplementary Material, further inquiries can be directed to the corresponding author/s.

## Ethics Statement

Written informed consent was obtained from the individual(s) for the publication of any potentially identifiable images or data included in this article.

## Author Contributions

All authors listed have made a substantial, direct and intellectual contribution to the work, and approved it for publication.

## Conflict of Interest

The authors declare that the research was conducted in the absence of any commercial or financial relationships that could be construed as a potential conflict of interest.

## Publisher's Note

All claims expressed in this article are solely those of the authors and do not necessarily represent those of their affiliated organizations, or those of the publisher, the editors and the reviewers. Any product that may be evaluated in this article, or claim that may be made by its manufacturer, is not guaranteed or endorsed by the publisher.
