# Supplementary material for: Using Generalizability Theory and Many-Facet Rasch Model to Evaluate In-Basket Tests for Managerial Positions
Source: Front Psychol. 2021 Jul 29;12:660553. doi: 10.3389/fpsyg.2021.660553 (PMC8359922; doi:10.3389/fpsyg.2021.660553)
Supplement: Supplementary file 1 [file Presentation_1.pdf]

Appendix A

The In-basket Tests Rating Scale

Using the scale provided, please indicate the degree to which you agree with each of the following statements regarding the candidate's performance in the ten in-basket tests (questions omitted; mark an answer for each item with "√").

1 = Disagree strongly

2 = Disagree

3 = Neither agree nor disagree

4 = Agree

5 = Agree strongly

Dimension 1: Planning ability items

Item number	Item	Score				
1	The candidate can process related official documents and information materials in an orderly manner.	1	2	3	4	5
2	The candidate can classify information based on its nature and characteristics.	1	2	3	4	5
3	The candidate has clear initiatives and action steps to identify objectives and organized ways to achieve goals.	1	2	3	4	5
4	The candidate has an effective timetable for action to complete the tasks.	1	2	3	4	5

Dimension 2: Communication and coordination

Item number	Item	Score				
5	The candidate can understand and show respect for the subordinates, listen to their opinions, coordinate various tasks and make things an organic whole.	1	2	3	4	5
6	The candidate can adjust the contradictions and conflicts in different interest groups using certain principles.	1	2	3	4	5
7	The candidate can perceive (identify) the causes of the problem, and grasp the connections among the related problems.	1	2	3	4	5
8	The candidate can form a correct judgment, and foresee the possible consequences of the problem.	1	2	3	4	5

Dimension 3: Capital operation and management

Item number	Item	Score				
9	The candidate can be aware of the connections between different information.	1	2	3	4	5
10	The candidate can make effective use of human, financial, physical and information resources.	1	2	3	4	5
11	The candidate can organize and distribute work loads/units systematically.	1	2	3	4	5
12	The candidate can sum up information and problems about management in an effective manner.	1	2	3	4	5

Dimension 4: Analysis and problem solving

Item number	Item	Score				
13	The candidate can analyze events in a quick, independent manner.	1	2	3	4	5
14	The candidate can propose and implement effective measures to solve problems in a clear logical, timely manner.	1	2	3	4	5
15	The candidate can make decisions in a timely and decisive manner even in an uncertain situation.	1	2	3	4	5
16	The candidate has a good, overall ability of analyzing and solve problems.	1	2	3	4	5

Dimension 5: Empowerment and controlling

Item number	Item	Score				
17	The candidate can motivate the subordinates and assign subordinates with tasks commensurate with their duties and expertise.	1	2	3	4	5
18	The candidate can provide the subordinates with clear directions for their actions and efforts.	1	2	3	4	5
19	The candidate can initiate, promote or terminate related work in a timely manner.	1	2	3	4	5
20	The candidate can maintain the normal operation of the organization, supervise and control expenses and other resources.	1	2	3	4	5

Appendix B

公文筐测试评定量表

指导语：请使用以下提供的量表，说明您对候选人在十项公文筐测试中表现的陈述的同意程度（问题省略；在每个项目的答案上打“√”）。

1=强烈不同意

2=不同意

3=既不同意也不反对

4=同意

5=强烈同意

序号	题目	计分				
1	能够有条不紊地处理各种公文和信息材料	1	2	3	4	5
2	能够根据信息的性质对信息进行分类处理	1	2	3	4	5
3	能够正确安排行动目标并制定有效的行动举措及步骤	1	2	3	4	5
4	能够制定有效的行动时间表来完成任务	1	2	3	4	5
5	能够理解、尊重下属并有效协调下属之间的关系	1	2	3	4	5
6	能够按照原则要求调节各方利益	1	2	3	4	5
7	能觉察问题起因并把握相关问题的关系	1	2	3	4	5
8	能够形成正确判断并预见问题的可能后果	1	2	3	4	5
9	能够注意到不同信息之间的关系	1	2	3	4	5
10	能够有效地利用人、财、物和信息资源	1	2	3	4	5
11	能够系统地安排和分配工作	1	2	3	4	5
12	能够有效汇总管理信息并发现存在的问题	1	2	3	4	5
13	能够迅速、独立地分析事件的概况	1	2	3	4	5
14	能够有条理地提出问题并给出有效的解决方法	1	2	3	4	5
15	能够在情况不明朗情境下果断地做出决策	1	2	3	4	5
16	具有分析并解决问题的大局观	1	2	3	4	5
17	能够激励下属并给分派与其职责及专长相适应的任务	1	2	3	4	5
18	能够给下属指明行动和努力的方向	1	2	3	4	5
19	能够适时地发起、促进或终止有关工作	1	2	3	4	5
20	能够维护组织机构的正常运转并监督和控制经费开支等	1	2	3	4	5

Appendix C

Now imagine that you are Shangyou Gao. You were promoted to the general manager of Company A one week ago.

Company A is an electronic technology company with 6 wholly-owned subsidiaries and more than 3,000 employees. The current management efficiency of the company is low, and the innovation ability is insufficient. The production efficiency has been declining recently.

Today is Monday, January 1, 2018, and it is 9 o'clock in the morning. Secretary Zhang has put the relevant documents in your document basket (a total of 10 documents). You must deal with all documents within two hours and ensure that all issues can be properly handled before January 31, 2018. Unfortunately, your phone is malfunctioning, and the company's internal LAN will not be able to be restored until tomorrow morning. Now please write down how you would solve the problems described in each of these documents.

Please note:

(1) Some documents and manuscripts should be solved immediately, and you should explain why they should be dealt with that way; some may not be solved immediately and should be forwarded to other departments for further solutions. However, you should provide appropriate suggestions and relevant reasons as future reference for the decision-makers to follow up.

(2) Please keep in mind that all tasks need to be completed within 2 hours!

(3) Please write down your answers on the answer booklet. You will receive no points if you write your answers on the question book.

FILE 1

Hello, Mr. Gao!

We have reached the end of the year. The annual bonus plan has not yet been determined. It has been a tradition of our company to pay an additional salary of one month and a half to employees at the end of each year. However, this year's economic situation is so severe and the company's efficiency is not good. It is possible that only half a month's salary could be provided this year, which is far from what employees would expect. If it is not handled properly, the company would lose a large number of key employees early next year. The vice president of Administration Department has just resigned, so we don't know how to deal with this situation. We look forward to your instructions!

xx Li, Human Resources Department
December 26, 2017

FLFE 2

Hello, Mr. Gao!

This year's consolation program for retired employees is now underway, and we have drawn up a preliminary activity plan, which is attached to the letter for your review. In addition to distributing corresponding gifts before the Spring Festival, we are also planning to hold a consolation party sometime in late January. We will invite

all senior leaders and more than 50 seniors to attend this event. We look forward to your comments on the activity plan!

xx Chen, Human Resources Department
December 26, 2017

FILE 3

Hello, Mr. Gao!

The Human Resources Department suggests that the Technology Department should provide relevant training for those technical staff to visit Germany. However, the Finance Department does not agree to provide training fees on the grounds that no prior plans or budgets were available for this training. After several rounds of negotiations, we have failed to come up with a solution in this regard. What do you think we should do?

xx He, Technology Department
December 24, 2017

FILE 4

Hello, Mr. Gao!

Some time ago, according to your instructions, I conducted an investigation within the company to look into why we had seen a decrease in the company's efficiency. It is generally believed that the main reason for the decline in the company's efficiency is the lack of technological R&D and innovation capabilities. At present, the gap between our company’s technical strength and other competitors is clear and still widening. In the past few years, we have basically been led by our competitors in the development of new products. Our company has always adopted a more passive follow-up strategy, which has helped us to maintain stable market shares, but greatly restricted the company's overall development and future prospects. Currently, due to cost considerations, our company is reluctant to invest heavily in product innovation. However, if we do not become a technological leader, our development space will become smaller and smaller, and our brain drain is very serious in this case. As of this year, the turnover of key talents in the technical department has reached 60%. I sincerely hope that you could closely work with the Human Resources Department on the plans to attract top talents to our company and reform the incentive mechanisms!

xx Mo, Technical Department
December 24, 2017

FILE 5

Hello, Mr. Gao!

Corporate administrative affairs are becoming more and more complicated. The company’s current secretarial work is taken by part-time, low-quality administrative clerks. Except for the general manager, other senior leaders have no secretaries to assist, and, accordingly, many management tasks are often delayed. The Administration Department plans to recruit a total of 11 full-time secretaries with high

salaries for the vice presidents and senior executives at all levels. We hope that this could better serve senior executives at all levels, improve the efficiency and quality of corporate administrative work, and also improve the company's external image. Please give us your instructions!

xx Liu, Administration Department
December 25, 2017

FILE 6

Hello, Mr. Gao!

The Spring Festival is approaching, can you draft a personal greeting letter for the leaders of each subsidiary in accordance with the company's practices?

xx Zhen, Administration Department
December 29, 2017

FILE 7

Dear Mr. Gao，

We are writing to remind you that the bill (number 452436718785) we sent you on December 12, 2016 has not been paid in full. A copy of the bill is attached to the letter. According to the pre-arranged agreement, starting from the date this letter is sent, if you fail to settle the bill within 14 days, you will have to pay an additional 1% interest per day.

Company XX
December 13, 2017

FILE 8

Dear Mr. Gao，

My name is David, the technical director of Software Company XX. The trial operation of our customized human resource management software system for your company is underway. According to the agreement, the trial operation will be completed before December 20. Also, according to the contract, we have agreed to complete the training for users of your company before December. However, your company has been unable to arrange a time for training to be conducted, and for this reason, the supervisor of your company's Human Resources Department has refused to pay the remaining payment. We hope that we can meet you to discuss the payment of fees and other matters. Thank you.

Software Company XX
December 21, 2017

FILE 9

Dear Mr. Gao，

I am Jing Xue, director of the STAR Media Event Planning Department. Over the years, our Star Media has established a close partnership with your company and provided good services to your company. However, recently our harmonious and

cooperative relationship has undergone some changes, which makes me feel very distressed.

Last year, we reached an oral agreement with Mr. Wang from the marketing department of your company that after the contract expires at the end of 2017, you will continue to sign a cooperation agreement with us. But last week, when I called the credit management department to confirm the renewal, they replied: any contract promised by the former manager will be postponed indefinitely, and further notice is required. This makes me very puzzled. The former general manager, on behalf of your bank, reached an oral agreement with our company. Our company has already, partially fulfilled some itemsas listed in the contract. Your company's personnel changes have now seriously harmed our company's interests. Violating business ethics will also have a negative impact on your company's reputation.

We cherish the long-established good cooperative relationship with your company. We hope that you could help solve this current issue, so that we could continue to believe in the good reputation of your company, and also enable us to achieve a win-win situation in terms of interests.

STAR Media Corporation
December 23, 2017

FILE 10

Hello, Mr. Gao!

I have communicated with the supervisor who is responsible for the contract renewal of STAR Media. The main reason we refused to sign the contract with Star Media was that their planning level was not as good as before, and the effect of the events they planned was relatively mediocre. Actually, we are actively looking for more suitable partners. The current claim that the contract signing time is delayed is just a pretext, which could buy us more time to find a more suitable partner.

xx Cheng, Marketing Department
December 23, 2017

Appendix D

您是高尚友先生，一周前刚被提升为 A 集团有限公司总经理。

A 集团有限公司是一家电子科技公司，有 6 家全资子公司，正式员工 3000 余人，企业目前管理效率较低，创新能力不足，生产效益近期有不断滑坡的趋势。

今天是 2018 年 1 月 1 日，星期一，现在是早上九点，张秘书已经将有关文件放入您的公文筐（共计 10 份文件）。您必须在两个小时内处理完所有文件，并保证所有问题都能在 2018 年 1 月 31 日前得到妥善的处理。很不幸现在您的电话发生故障，而公司内部的局域网也要等到明天上午才能恢复使用。现在请您将您对处理意见写在每份文件下面。

请注意：（1）对于某些文件或者手稿，您需要做出相应的处理，并需要说明您这样处理的理由；即使碰到您不能马上决定的事情，或需要转发由其他多个部门协助处理的文件，也应当提供适当的建议和理由，以备决策者参考。（2）所有的任务需要在 2 小时内完成，请注意时间安排！（3）对每份文件的处理意见和理由都请写在答题册上，写在题本上不予给分。

FILE 1

高总，您好！

已到年末，年度奖金方案尚未确定。以往每年年底人均加发一个半月工资，已成传统。但今年经济形势严峻，公司效益不佳，只能加发半个月工资，这与员工的期望差距甚大。如处理不好，明年初骨干员工会大量流失。行政副总刚刚离职，我们不知如何应对？请您批示！

人力资源部李 xx

2017 年 12 月 26 日

FLFE 2

高总，您好！

新年度的退休员工慰问工作正在启动，我们已经初步拟好活动方案，并附在信件后面。除了春节前派发相应礼物外，我们还打算举办一个慰问晚会，届时将邀请各位高层与五十多名老前辈共同出席活动，时间在一月下旬。希望您过目活动方案！

人力资源部陈 XX

2017 年 12 月 26 日

FILE 3

高总，您好！

人力资源部建议我们对技术部门的人员进行德国出国的技术培训，但是财务部不同意给我们培训费用，理由是这项培训事先没有计划和预算。经过多番协商，双方都不能达成一致，您看该怎么处理？

技术部何 XX

2017 年 12 月 24 日

FILE 4

高总，您好！

前段时间我按照您的指示对企业内部进行一次调查，大家普遍认为导致公司效益下降的主要原因还是技术研发创新能力不足。目前我们的技术实力与竞争对手 相比已经有了明显的差距，这几年我们在新产品开发上基本都是被竞争对手牵着走，我们公司一直采取较为被动的跟随策略，这虽然能保持稳定的市场份额，但对公司的整体发展和未来前景限制很大。而我们公司目前出于成本考虑，也不敢贸然在产品创新上进行大投入，但如果不做技术的领先者，我们的发展空间会越来越小，从这些年公司的业务发展情况也可以看出这一点，而且在这种情况下我们的人才流失十分严重。直至今年，技术部骨干人才流失已达 60%。我真诚希望您能和人力资源部商量好人才引进计划和激励机制改革！

技术部莫 XX

2017 年 12 月 24 日

FILE 5

高总，您好！

企业行政事务日益繁杂，公司目前的文秘工作均由素质较低的行政文员兼做，除总经理外，其他高层领导均无秘书，很多管理工作经常被耽误。行政部计划为各位副总及各级高管高薪招聘共计 11 名专职秘书，以确保更好地服务于各级高管，提高企业行政工作效率和工作质量，并改进公司的对外形象。妥否？请您批示！

行政部刘 XX

2017 年 12 月 25 日

FILE 6

高总，您好！

春节将至，是否能根据公司惯例为各个子公司的领导写一封亲笔问候信？

行政部甄 XX

2017 年 12 月 29 日

FILE 7

尊敬的高先生：

该来信是提醒您，我们于 16 年 12 月 12 日寄去的编号为 452436718785 的账单尚未付清，随信寄去账单副本。根据事先达成的协议，以这封信寄出的日期算起，如果 14 日内未按协议结账，您将要加付每天 1%的利息。

XX 公司

2017 年 12 月 13 日

FILE 8

高总，您好！

我是 XX 软件开发公司的技术主管 David，我们为贵公司定制的人力资源管理软件系统的试运行正在进行。按照协议规定，在 12 月 20 日之前完成试运行。按照合同规定，我们曾商定过，12 月份前完成对贵公司使用者的培训。由

于贵公司一直无法安排时间进行培训，贵公司人力资源部主管人员以此为由，拒绝支付剩余款项。我们希望，能否与您见面协商一下费用的支付问题及其他事宜？谢谢您。

XX 软件公司

2017 年 12 月 21 日

FILE 9

尊敬的高总经理，您好：

我是 STAR 传媒活动策划部总监薛静。多年来，我们星空传媒与贵公司建立很好的合作伙伴关系，为贵公司提供了良好的服务。但最近我们融洽的合作关系发生了小小的变故，令我十分苦恼。

去年我们和贵公司的市场部王先生达成口头协议：2017 年底合同到期之后会继续和我们签署合作协议。但上周，我打电话到信贷管理部确认续签事宜时他们却答复我：有关前任经理承诺的任何合同事宜都将无限期延期签署，需等待进一步通知。这令我产生很大的疑惑，前任总经理是代表贵行与我司达成的口头协议，我司已经部分履行合同中的事项，但如今贵公司的人事变动严重损害我公司的利益，这种违反商业道德的行为会给贵公司的声誉带来负面的影响。

我们很珍惜和贵公司长久以来建立的良好合作关系，希望高经理能够帮助我们解决目前的这个疑惑，让我们能够继续坚信贵公司的良好信誉，也能让我们双方达到利益上的共赢。

STAR 传媒公司

2017 年 12 月 23 日

FILE 10

高总，您好！

我已经和负责 STAR 传媒合同续签的主管做了沟通，我们拒绝和星空传媒签约的主要原因是他们的策划水平大不如前，活动效果很一般。我们正在积极的寻找更合适的合作伙伴。目前对他们宣称合同签约时间推迟，只是一个托词，这能为我们找到更合适的合作伙伴争取时间。

市场部成 XX

2017 年 12 月 23 日

Table 1

Minimums, Maximums, Means, Standard Deviations, and Correlations for Five Dimensions

	Descriptive Statistics					Correlations				
Variable	N	Min	Max	Mean	SD	1	2	3	4	5
1. Planning	78	5.00	20.00	16.08	3.07	--				
2. Communication and coordination	78	5.00	20.00	15.53	3.05	0.58**	--			
3. Capital operations and management	78	4.00	20.00	16.58	3.69	0.56**	0.61**	--		
4. Analysis and problem-solving	78	4.00	20.00	14.80	3.82	0.57**	0.65**	0.64**	--	
5. Empowerment and controlling	78	4.00	20.00	15.11	3.68	0.69**	0.61**	0.62**	0.63**	--

Note. 1= Planning, 2= Communication and coordination; 3= Capital operations and management, 4= Analysis and problem-solving, 5= Empowerment and controlling. ** Correlation is significant at the 0.01 level (2-tailed).

Table 2

G Study

Effect	df	MS	Variance component	percentage of total variance
persons (p)	77	73.19	2.61	53.22%
items (i)	4	89.09	0.21	4.23%
raters (r)	4	62.08	0.16	3.22%
pi	308	7.84	1.48	30.09%
pr	308	0.45	0.00	0.01%
ir	16	0.41	0.00	0.02%
pir,e	1232	0.45	0.45	9.24%

Table 3

D Study

Raters	Items	Variance Component of Persons	Norm-referenced Test	Criterion-referenced Test
			G-coefficients	Dependability coefficients
5	7	2.61	0.92	0.90
4	7	2.61	0.92	0.90
3	7	2.61	0.92	0.89
2	7	2.61	0.92	0.88
1	7	2.61	0.90	0.85
5	6	2.61	0.91	0.89
4	6	2.61	0.91	0.89
3	6	2.61	0.91	0.88
2	6	2.61	0.90	0.87
1	6	2.61	0.89	0.84
5	5	2.61	0.89	0.87
4	5	2.61	0.89	0.87
3	5	2.61	0.89	0.86
2	5	2.61	0.89	0.85
1	5	2.61	0.87	0.82
5	4	2.61	0.87	0.85
4	4	2.61	0.87	0.84
3	4	2.61	0.87	0.84
2	4	2.61	0.86	0.82
1	4	2.61	0.84	0.79
5	3	2.61	0.83	0.81
4	3	2.61	0.83	0.80
3	3	2.61	0.83	0.80
2	3	2.61	0.82	0.79
1	3	2.61	0.80	0.75

Table 4

FACETS Analysis of Candidates' Ability

Partici pant	Abilit y	Error	Infit MS	Partici pant	Abilit y	Error	Infit MS	Partici pant	Abilit y	Error	Infit MS
1	-0.72	0.15	1.41	27	0.32	0.16	1.02	53	0.66	0.17	3.86
2	0.60	0.16	0.40	28	1.95	0.21	2.13	54	-0.25	0.15	1.46
3	1.31	0.19	0.52	29	0.11	0.15	1.53	55	-0.82	0.15	0.22
4	0.50	0.16	0.56	30	-0.25	0.15	1.28	56	-1.26	0.15	0.72
5	0.11	0.15	0.74	31	1.20	0.18	0.87	57	-1.48	0.15	1.61
6	1.04	0.18	0.78	32	1.24	0.19	0.56	58	-0.18	0.15	0.59
7	0.47	0.16	0.64	33	-1.87	0.16	0.25	59	0.27	0.15	1.21
8	-0.55	0.15	0.86	34	-0.82	0.15	0.23	60	0.11	0.15	0.51
9	0.91	0.17	0.57	35	-0.80	0.15	1.32	61	-1.87	0.16	0.39
10	1.01	0.18	0.91	36	0.85	0.17	0.48	62	-0.95	0.15	0.42
11	2.58	0.25	0.72	37	0.91	0.17	0.78	63	0.25	0.15	3.64
12	0.35	0.16	0.34	38	-1.99	0.16	0.37	64	-1.34	0.15	0.27
13	0.55	0.16	0.41	39	-0.20	0.15	0.41	65	0.02	0.15	1.37
14	2.24	0.23	0.63	40	-1.32	0.15	4.57	66	-0.20	0.15	0.63
15	0.20	0.15	1.12	41	-1.70	0.15	0.59	67	1.27	0.19	0.46
16	1.24	0.19	1.36	42	-0.67	0.15	2.48	68	0.23	0.15	0.66
17	2.04	0.22	1.63	43	-1.41	0.15	0.24	69	-1.23	0.15	0.26
18	2.13	0.22	1.24	44	-1.50	0.15	3.39	70	-0.61	0.15	0.84
19	0.83	0.17	0.73	45	0.45	0.16	0.46	71	-1.19	0.15	0.20
20	0.25	0.15	0.39	46	-1.26	0.15	0.21	72	0.77	0.17	0.79
21	0.98	0.18	0.93	47	0.55	0.16	0.46	73	-1.19	0.15	0.37
22	1.69	0.20	0.79	48	-1.28	0.15	1.18	74	-0.05	0.15	2.21
23	-0.22	0.15	1.79	49	-1.00	0.15	1.40	75	-0.67	0.15	1.27
24	0.27	0.15	0.65	50	0.23	0.15	0.76	76	-1.21	0.15	0.55
25	-0.05	0.15	0.41	51	0.63	0.16	0.58	77	-0.95	0.15	1.44
26	-0.37	0.15	0.99	52	0.32	0.16	1.52	78	-0.16	0.15	0.50

Note. Separation Reliability=0.98, chi-square=2864.0, df=77, significance=0.001, Infit SD=0.84

Table 5

FACETS Analysis of Item Difficulty

Item		Difficulty	Error	Infit MS
1.	Planning	0.50	0.04	2.17
2.	Communication and coordination	-0.12	0.04	0.56
3.	Capital operations and management	-0.05	0.04	0.89
4.	Analysis and problem-solving	-0.07	0.04	0.61
5.	Empowerment and controlling	-0.26	0.04	0.65

Note. Separation Reliability=0. 98, chi-square=3.9, df=4, significance=0.27, Infit SD=0.68

Table 6

FACETS Analysis of Raters' Severity

Rater	Severity	Error	Infit MS
1	-0.57	0.04	0.88
2	-0.55	0.04	1.03
3	-0.33	0.04	0.91
4	-0.73	0.04	1.16
5	-1.01	0.04	1.01

Note. Separation Reliability=0. 79, chi-square=154.1, df=4, significance=0.001, Infit SD=0.11

Table 7

Bias Analysis of Raters and Candidates

Rater	Participant	Assessment Ability	Expected Ability	Bias	SE	T
2	21	0.52	1.80	-1.28	0.61	-2.08

```
-----------------------------------------------------------------------------------------------------------
|Measure|+participants       |-items                                      |+participants|-Rater          |ZHNL |
-----------------------------------------------------------------------------------------------------------
+   3 +                     +                                            +             +                +(10) +
|     |                     |                                            |             |                |     |
|     |                     |                                            |             |                |  9  |
|     |                     |                                            |             |                |     |
|     | 11                  |                                            | *           |                |     |
|     |                     |                                            |             |                |     |
|     |                     |                                            |             |                |     |
|     |                     |                                            |             |                |     |
|     | 14                  |                                            | *           |                | --- |
|     | 18                  |                                            | *           |                |     |
+   2 + 17                  +                                            + *           +                +     +
|     | 28                  |                                            | *           |                |     |
|     |                     |                                            |             |                |  8  |
|     | 22                  |                                            | *           |                |     |
|     |                     |                                            |             |                |     |
|     |                     |                                            |             |                |     |
|     |                     |                                            |             |                | --- |
|     | 3  67               |                                            | **          |                |     |
|     | 16 31 32            |                                            | ***         |                |     |
|     |                     |                                            |             |                |  7  |
+   1 + 6  10 21            +                                            + ***         +                +     +
|     | 9  36 37            |                                            | ***         |                |     |
|     | 19 72               |                                            | **          |                | --- |
|     | 53                  |                                            | *           |                |     |
|     | 2  51               |                                            | **          |                |     |
|     | 4  7  13 47         | Planning(1)                                | ****        |                |  6  |
|     | 45                  |                                            | *           |                |     |
|     | 12 24 27 52 59      |                                            | *****       |                |     |
|     | 15 20 50 63 68      |                                            | *****       |                | --- |
|     | 5  29 60            |                                            | ***         |                |     |
*   0 * 25 65 74            *                                            * ***         *                *  5  *
|     |                     | Analysis(4) Communication(2)  Management(3)|             |                |     |
|     | 23 30 39 54 58 66 78|                                            | *******     |                |     |
|     |                     | Controlling(5)                             |             | rater3         | --- |
|     | 26                  |                                            | *           |                |     |
|     | 8                   |                                            | *           |                |     |
|     | 70                  |                                            | *           | rater1  rater2 |  4  |
|     | 1  42 75            |                                            | ***         | rater4         |     |
|     | 34 35 55            |                                            | ***         |                | --- |
|     |                     |                                            |             |                |     |
+  -1 + 49 62 77            +                                            + ***         + rater5         +     +
|     |                     |                                            |             |                |  3  |
|     | 69 71 73 76         |                                            | ****        |                |     |
|     | 40 46 48 56 64      |                                            | *****       |                |     |
|     | 43                  |                                            | *           |                | --- |
|     | 44 57               |                                            | **          |                |     |
|     |                     |                                            |             |                |     |
|     | 41                  |                                            | *           |                |     |
|     |                     |                                            |             |                |  2  |
|     | 33 61               |                                            | **          |                |     |
+  -2 + 38                  +                                            + *           +                + (0) +
-----------------------------------------------------------------------------------------------------------
|Measure|+participants       |-items                                      | * = 1       |-Rater          |ZHNL |
-----------------------------------------------------------------------------------------------------------
```

Figure 1. Variable map of all facets
